# Spatial and Temporal Data Visualisation for Mass Dissemination: Advances in the Era of COVID-19

**DOI:** 10.3390/tropicalmed8060314

**Published:** 2023-06-09

**Authors:** Archie C. A. Clements

**Affiliations:** 1 Peninsula Medical School, University of Plymouth, Plymouth PL4 8AA, UK; archie.clements@plymouth.ac.uk; 2 Telethon Kids Institute, Nedlands, Perth, WA 6009, Australia

**Keywords:** COVID-19, spatial data, temporal data, data visualization, dashboard

## Abstract

The COVID-19 pandemic is the first major pandemic of the digital age and has been characterised by unprecedented public consumption of spatial and temporal disease data, which can enable greater transparency and accountability of governments to the public for their public health decisions. A variety of state and non-state actors have collated and presented maps, charts, and plots of data related to the pandemic in both static and dynamic formats. In particular, there has been a proliferation of online dashboards presenting data related to the pandemic. The sources and types of information displayed have evolved rapidly during the pandemic, with a general trend towards providing more specialised information pertinent to specific aspects of epidemiology or disease control, as opposed simply to disease and death notifications. Limited evaluation of the quality of COVID-19 data visualisation tools has been conducted and significant effort now needs to be spent on standardisation and quality improvement of national and international data visualisation systems including developing common indicators, data quality assurance mechanisms and visualisation approaches, and building compatible electronic systems for data collection and sharing. The increasing availability of disease data for public consumption presents challenges and opportunities for government, media organisations, academic research institutions, and the general public. A key challenge is ensuring consistency and effectiveness of public health messaging to ensure a coordinated response and public trust in intervention strategies. Capitalising on opportunities for greater government accountability for public health decision-making, and more effective mobilisation of public health interventions, is predicated on the provision of accurate and timely information.

## 1. Introduction

The current COVID-19 pandemic is the first major human pandemic (causing over 1 million deaths) since computers and the internet became available to the majority of the world’s population. In the new information age, health data have become more accessible to the general public than ever, and this is particularly true with respect to the COVID-19 pandemic. To present data in a way that is engaging and of value for public consumption, a plethora of individuals and organizations have created COVID-19 data visualization platforms or data representations. Identification of spatial and temporal patterns of disease is particularly important for the understanding and interpretation of disease risk, both by health decision-makers and the general public. Governments, international organizations, and the private sector have made large volumes of information related to the COVID-19 pandemic partially or fully available, underpinning the global effort to provide transparent and accessible spatial and temporal disease data [1], referred to as the *democratization of data* and a *revolution in disease reporting* by Koch [2].

The collation and updating of COVID-19 data for public dissemination is a major undertaking, given the disparate sources from which the data are extracted. Groups collating data for public dissemination include Governments of many countries and regions, academic organizations, academic-government collaborations [3,4], academic-private sector collaborations [5], international agencies such as the World Health Organization [6], news media organizations and publishers such as the New York Times and The Atlantic, corporate entities such as Worldometer [7] and Deloitte [8], and not-for-profit organizations such as Our World in Data [9]. These groups have their own online data visualisation platforms that can be accessed by the public, whilst many other organizations that present COVID-19 data also extract data from these sources [10,11]. Indeed, there has been a significant amount of recycling and assemblage of data from overlapping sources, with the outputs varying slightly between different platforms depending on the data sources used and processing and presentation decisions made. 

Here, the sources of data, approaches to spatial and temporal data representation and strengths and weaknesses of the approaches taken are evaluated and discussed. The purpose of this review is to document and analyse ways in which spatially and temporally referenced data have been made available to decision-makers, health professionals, and the public through online and other means of mass dissemination. The trade-off between greater transparency of current situations to the general public and accountability of decision-makers on the one hand and the need for careful curation of data and controlled public health messaging for the public good on the other is discussed with reference to the perspectives of different groups affected by pandemics.

## 2. Platforms for Information Display

**Figure 1:**
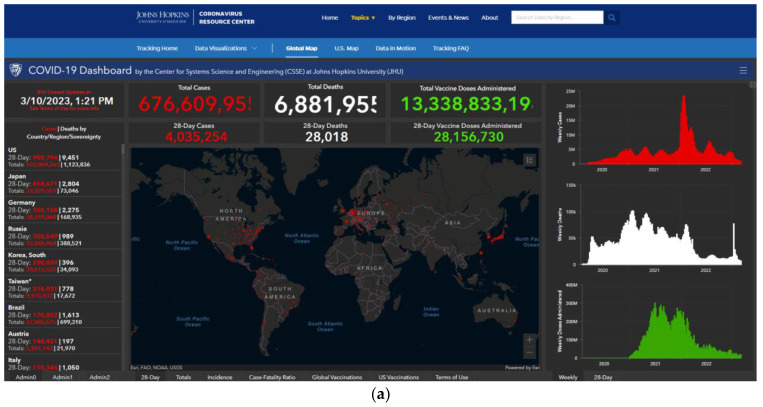

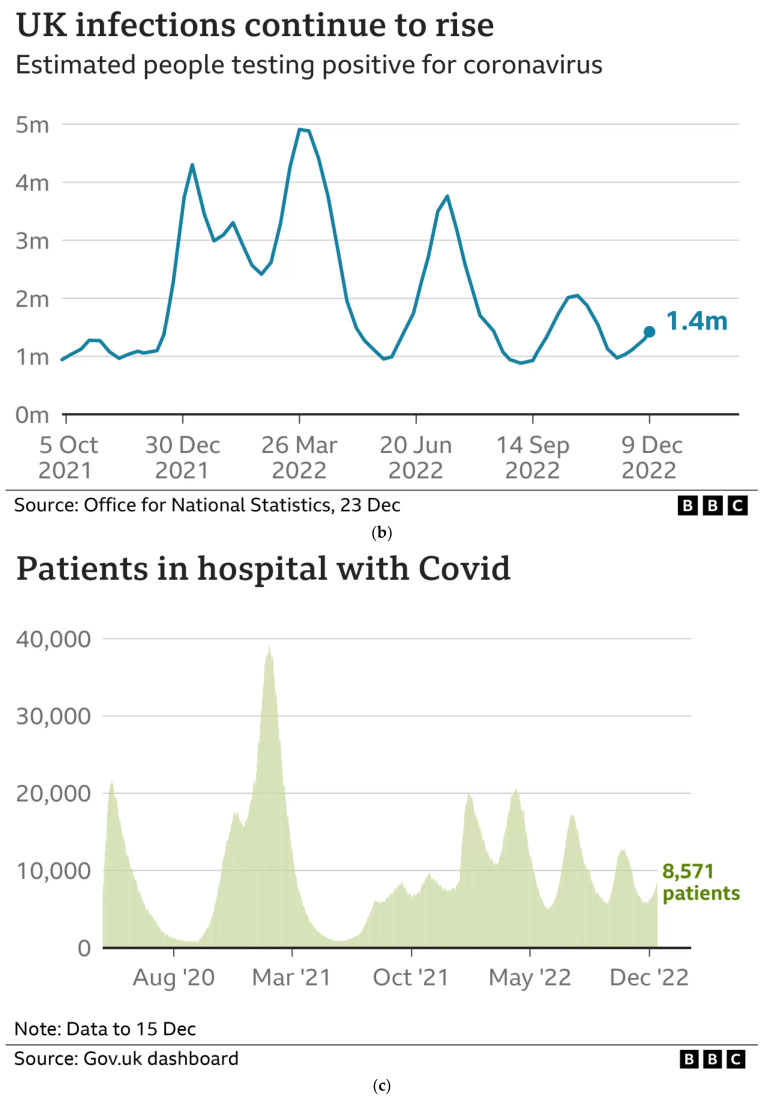

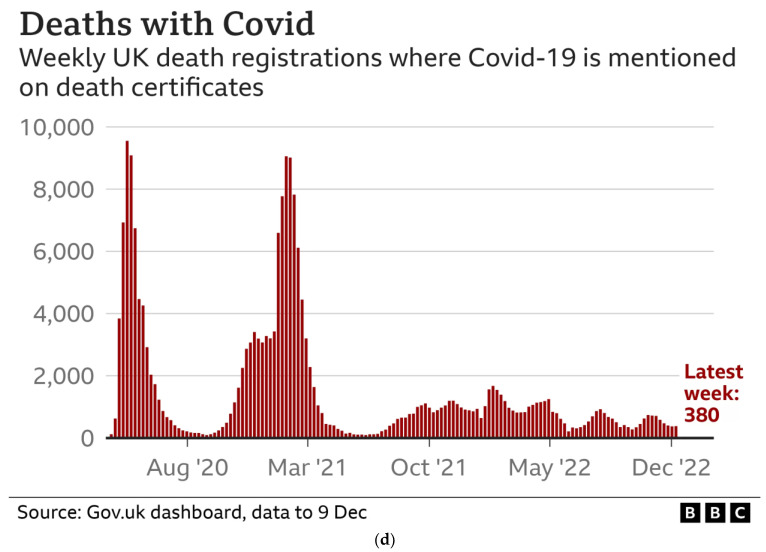
Examples of (**a**). a COVID-19 dashboard user interface, the Johns Hopkins University COVID-19 Dashboard (source: https://coronavirus.jhu.edu/map.html, accessed on 11 April 2023) and (**b**–**d**). static representation of data time-series using histograms and line graphs by a news media organization, the British Broadcasting Corporation (source: https://www.bbc.co.uk/news/uk-51768274, accessed on 11 April 2023).

Organizations have presented spatial and temporal data in a variety of ways including static presentation of data and via dynamic, online platforms, or dashboards (Figure 1; Table 1). Online dashboards are highly interactive, allowing the user to select variables of interest, plot, or map them in different formats and drill down to particular levels of aggregation. Many countries around the world present official COVID-19 related data via online dashboards (for a list of European data repositories and dashboards, see [12]). In many instances, online dashboards have been sources of data for scientific research, but they have also presented data using visualisation methods such as graphs of epidemic curves and maps that have been prepared for consumption by the general public and public health decision-makers [13]. Kolak and colleagues [5] described a participatory approach to developing a COVID-19 dashboard, where multiple stakeholders were invited to contribute to the development of the dashboard to ensure it met consumer needs.

Most dashboards have presented epidemiological information on cases and deaths, whilst some provided additional information on hospitalizations, intensive care unit admissions, ventilator use, testing coverage, and recovered and active cases. A small number of dashboards provided more specialised or contextual information. As the pandemic has progressed, spatial visualisation platforms have become more diverse and specialised, ranging from aggregate mapping of cases to the mapping of exposure sites [14], population mobility [15], urban environmental suitability for social distancing [1], morgue capacity [16], risk of outbreaks for events of different sizes [17], and personal risk of infection [18]. Dashboards have also utilised simple analytical approaches to provide estimates of risk including automated alerts for locations of interest [14] and cluster detection [5]. Following the discovery and distribution of vaccines, dashboards increasingly focussed on presenting data related to vaccination rollout and coverage, with health system variables being added to online platforms that initially focused on presenting epidemiological information [19].

## 3. Sources of Data

Most online platforms have integrated data from multiple streams including data on COVID-19 cases and deaths, census and other forms of population data, and other, more specialised data sources such as vaccination registers, hospital admissions data and other patient records, medical laboratory data, and adjunct sources of information such as human mobility tracking and weather data. Data democratization has meant that a wider variety of data sources have been accessed. For example, some organizations have made use of twitter feeds, news reports, and direct communication from contributors, in addition to more traditional sources such as official government reports [5,20]. 

Official data from Governments are generated from multiple data sources, including health centres, testing laboratories, and death records, and rely on timely and accurate data reporting systems [3]. One of the most widely cited platforms is the Johns Hopkins COVID-19 dashboard [20] (Figure 1a). In the early stages of the pandemic, operatives entered data into the database underlying the dashboard manually, but as the volume of data and variety of sources of information increased, data extraction became semi-automated. 

Several data scraping toolsets linked to public dashboards have been reported including the COVID-19 European Regional Tracker [12], CRISPER from Australia [14], and the COVID-Scraper, which presents spatiotemporal data from 58 countries [21]. The COVID-Scraper is an open-source toolset for the extraction, processing, storage, and dissemination of spatiotemporal data related to the COVID-19 pandemic. Such approaches enable the collation of data from sources that are both digitally structured (e.g., tables, spreadsheets, and databases) and unstructured (e.g., text and figures). 

## 4. Access Control for Data Visualisation Platforms

An important aspect of platform configuration is who is provided access to information; this influences the extent to which information can be democratized and also the level of control that can or should be exercised to ensure the protection of rights (e.g., privacy) and to ensure good decision-making. The configuration of dashboards for mobile telephone use has ensured more widespread access to COVID-19 data, reflecting contemporary preferences for public consumption of information [22]. However, some organizations make online data visualisation platforms only available only by paid subscription (such as the New York Times and The Telegraph) or limit access to authorized individuals due to the sensitive nature of the data (such as the COVER-NC vaccine equity dashboard [23]). Interestingly, the COVID-19 scraper is openly reported to have functions to bypass mechanisms for restricting data access [21], thereby allowing wider dissemination of information that some official bodies consider sufficiently sensitive to restrict. Choice of language is an important aspect of accessibility—the Japan LIVE dashboard is available in Japanese, Chinese, and English [24], opening access to a wider international audience. 

Privacy is an important consideration in identifying the most appropriate minimum level of data aggregation [3]. Various approaches have been proposed for maintaining data privacy in COVID-19 dashboards, with differential privacy mechanisms (whereby small amounts of variation are added to key variables to obscure identifying information) being one such approach [14]. 

## 5. Types of Data Representation

Time-series plots showing raw data, often overlaid with smoothing functions (to separate and visualise underlying temporal patterns from background noise), are the most common way of demonstrating temporal data, at sub-national, national, and global levels. Time series are presented on linear and logarithmic scales, the latter allowing for easier visual comparison of sets of observations where there are extreme outliers (i.e., very large numbers of cases in some places or times, as can happen in large outbreaks, that would make visualisation of differences in non-outlier places and times difficult) and for comparing rates of change (e.g., the speed of propagation of an epidemic in one place or time compared to others, which was particularly useful in the early, exponential phase of the pandemic and during subsequent waves). Data have included raw counts and counts normalized by population size, allowing visualisation of variation in risk or coverage of testing and vaccination.

Choropleth maps are the most common format for spatial data visualization, whereby areas (e.g., sub-national administrative units, or countries) are coloured according to the value of the variable of interest [25]. Choropleth maps are commonly used because surveillance data are typically reported by areal administrative units, rather than by an individual’s address. In the case of COVID-19, the variables mapped could be the number of cases or the incidence of infection, although the latter is more effective in communicating risk (whereas the former is distorted by the size of the population in each area). 

All of the inherent problems of choropleth maps apply to COVID-19 data displays, including the fact that observed patterns can be influenced by the shape and size of the areas on the map (whereby large areas dominate the map), and the level of aggregation at which the data are presented (whereby high levels of aggregation can mask important spatial variation, for example, the presence of disease clusters and low levels of aggregation can result in unstable estimates due to small numbers) [25,26]. The level of data aggregation can also affect the utility of the map for public health decision-making, particularly if it is too crude to inform local interventions [27]. Classification schemes (whereby continuous or ordinal variables such as case numbers or disease incidence are categorized into discrete classes for the purpose of visual display) can also be highly influential on the appearance of the map and visual interpretation of the data, because different schemes visually emphasise different parts of the distribution of the variable being mapped (e.g., the tails versus the central area), which can in turn impact on overall interpretation of risk and influence decisions on an individual’s or organization’s response [25,26]. Other cartographic elements such as the colour scheme, the inclusion of geographical features to enable spatial referencing of the data, scale bars, legends, and overlays of different variables can further influence the appearance and interpretation of maps [26]. 

Evolution of the spatial distribution of the pandemic can be presented over time using panels of choropleth maps or as spatial data animations (e.g., [12]). Many dashboards allow for customisation by users of visual displays including time-series charts and maps [5].

## 6. Data and Visualisation Platform Quality

The quality of the data feeding into the maps and charts has been an important and much-commented-upon issue throughout the pandemic. The biggest issue has been under-reporting [10], primarily caused by the slow take-off and inconsistent application of testing in many countries around the world. Interpretation of data has been made more challenging due to inconsistent and changing case definitions, both with respect to infections and deaths. Changes in the way that data are reported can also create challenges. For example, the European Centre for Disease Prevention and Control has shifted from daily to weekly and back to daily reporting at various stages of the pandemic [12]. Other challenges include a lack of data permanence, language, and regional date formatting differences in source data, missing data (and different ways of handling missing data between data sources), and temporal lags in data availability at different levels [28].

Few studies have evaluated the quality of temporal and spatial COVID-19 data visualisation platforms. Zhao and colleagues [29] conducted a content analysis of dashboards from Japan, China, and South Korea, comparing the number of indicators in each dashboard, and finding the numbers of indicators to be broadly similar. However, they did not undertake a detailed evaluation of quality. The most comprehensive evaluations to date of multiple spatial and temporal data visualisation platforms have been conducted by Ivanković and colleagues [11] and Monkman and colleagues [30]. Both of these evaluations pertained specifically to online dashboards. 

Ivancović and colleagues focussed on actionability, which they summarized according to seven features: (1) knowing and clearly stating the desired consumers of the information; (2) selection and presentation of appropriate indicators; (3) clearly stating the sources of data and methods used to generate indicators; (4) demonstrating variation over time and linking changes to public health interventions; (5) providing as high a spatial resolution as possible to enable consumers to evaluate local risk; (6) disaggregating data to population sub-groups to further enable evaluation of risk; (7) providing narrative information to enhance interpretation of the data by the consumer. Overall, they found only 20/158 dashboards to achieve the maximum score for actionability. Clearly, a major emphasis of their evaluation was on maximising transparency and rewarding dashboards that best assisted consumers to reach appropriate conclusions from the data. Using a similar approach and criteria, Bos and colleagues [19] undertook a detailed evaluation of a single dashboard, the Dutch national COVID-19 dashboard, with a focus on its development and evolution at three time points during the first year of the pandemic. They reported that the number of indicators increased over time, and displays became customizable and more detailed, with the target audience shifting from experts and policy makers to the general public. 

Monkman and colleagues used an established heuristic for evaluating dashboard visualization [31], which included ten criteria: (1) visibility of system status; (2) match between the system and the real world (i.e., avoidance of the use of technical jargon or variable names that are difficult to understand in real-world decision making); (3) user control and freedom; (4) consistency and standards; (5) recognition rather than recall; (6) flexibility and efficiency of use; (7) aesthetics and design; (8) spatial organization; (9) information coding; (10) orientation. They made some criticisms of COVID-19 dashboards, including that they used technical jargon and undefined or unclear labels, and they presented a lack of denominator information and numbers with excessive precision, there was a lack of standard designs across jurisdictions of the same country, and displays exceeded the capacity of single screens, requiring users to scroll and recall information or had a busy and disorganized layout, and there was a general lack of information about data sources. 

Few other examples of formal evaluations have been reported with respect to accuracy, reliability, or utility. One study involved qualitative evaluation of a specialised system, although it did not use a highly structured methodological approach [16]. Another study investigated access to a COVID-19 data visualization platform in the Czech Republic using Google Analytics [4] and it found that the majority of visitors used a mobile device or tablet, with the Google Chrome web browser, and over 80% of visitors were return visitors. Such information on patterns of use and access is important for the design of future online data visualization platforms, particularly with respect to the design of charts and maps to ensure that are easily viewed on a small-screen format.

## 7. Discussion and Conclusions

Given that each dashboard uses different sources of data, the information presented for a specific time-point and jurisdiction can vary slightly and this can become an unhelpful focus of the response to the data, rather than the patterns of risk that the dashboards are presenting. If online data visualisation platforms are able to provide transparent and reliable information, they can be helpful in mitigating against the massive amounts of misinformation that is spread during major global events such as the COVID-19 pandemic [32]. However, with the ever-increasing accessibility of the software required to produce maps, charts, and other forms of data visualization, more people are able to generate and distribute spatial and temporal information online [27]. Additionally, those who produce the maps and charts can lose control of their use once they are in the public domain, potentially leading to widespread misinterpretation. Publicly available spatial data are particularly vulnerable to being co-opted and misused by conspiracists to promulgate spurious theories about disease aetiology, exposure, and public health interventions, with potentially harmful consequences for public health, as has been the case with COVID-19 [33].

Perhaps the biggest change with the current pandemic compared to previous pandemics, leading to data democratization [2], has been the role of non-state actors in generating and distributing vast quantities of spatial and temporal information about the pandemic to the general public. This comes with significant risks, but also significant benefits. Variations from official statistics, and between the statistics reported by different non-state actors, can create confusion and mistrust. Quality assurance practices have varied significantly, and limited evaluation of spatial and temporal data platforms has been reported, raising questions about the quality of the information provided. Undoubtedly, the openness of access to disease data creates significant challenges for governments in managing public messages regarding the pandemic and acting as a single source of truth. 

However, access to and dissemination of data by non-state actors can lead to greater transparency and accountability of governments for the decisions that they make. Attempts by governments to suppress information, or manipulate information for political purposes, were undermined by the open availability of data and information related to the current pandemic. Public interest in, and understanding of, epidemiological concepts such as epidemic curves, epidemic waves, reproductive rates, spatial heterogeneity in disease risk, and spatial inequity in healthcare access were all enhanced by open access to disease data. This greater literacy with respect to epidemiological concepts could be harnessed to ensure a better coordinated and effective public response to future pandemics. 

Open access to disease data is only likely to increase, despite efforts by some governments to control access and limit the availability of information for reasons that might or might not be in the public good, however, that is defined. Challenges for the next pandemic vary according to perspective: for example, for governments, the challenge will be to harness the benefits of open access through collaborating with, funding, and supporting non-state actors with the technical expertise and infrastructure to support the dissemination of high-quality information to the public, whilst maintaining influence over public health messaging to ensure a cohesive response by the general public. Appropriate legislation, regulation, and enforcement will need to be developed to protect the rights of individuals with respect to privacy and enable access to information that is accurate and necessary. Additionally, for low-resource settings, international support will be required to ensure adequate investment in technology and high-functioning systems, including human capacity, for disease reporting. For international organizations, the political dimensions of data sharing and cross-jurisdictional transparency will need to be skilfully managed. 

For media organisations (including traditional and social media), the challenge will be to balance their social responsibility to provide accurate information in a way that is constructive and supports the public good, whilst maintaining independence, holding decision-makers to account, and pursuing their commercial interests. For academic institutions, the challenges include being responsive and mobilising resources (particularly intellectual and technical resources) in a sufficiently timely manner to be of assistance to governments and the public, to learn how to respond to the needs of non-academic partners, and to continue to research and develop new and optimal methods for information generation and dissemination. The latter will require multi-disciplinary approaches that might include data and computer scientists, epidemiologists and public health researchers, clinical researchers, operational researchers, decision scientists, design experts, social scientists, political scientists, and implementation scientists. For the general public, the challenge will be to develop strategies for selecting, assessing and responding to relevant information from the overwhelming, and growing body of information available to them.

## Figures and Tables

**Table 1:** Examples of different types of COVID-19 spatial and/or temporal data visualisation tools for mass consumption.

Name	Website	Organization
Dashboards *		
WHO Coronavirus (COVID-19) Dashboard	https://covid19.who.int/ (accessed 11 April 2023)	World Health Organization
COVID-19 Dashboard by the Center for Systems Science and Engineering (CSSE) at Johns Hopkins University (JHU)	https://coronavirus.jhu.edu/map.html (accessed on 11 April 2023)	Johns Hopkins University
How’s My Flattening	https://howsmyflattening.ca/#/home (accessed on 4 April 2023)	University of Toronto and partners
COVID Act Now	https://covidactnow.org/?s=1710634 (accessed on 4 April 2023)	Georgetown, Stanford and Harvard Universities
1Point3Acres	https://coronavirus.1point3acres.com/en (accessed on 6 April 2023)	
COVID-19 Info Switzerland	https://corona-data.ch/ (accessed on 4 April 2023)	Swiss National COVID-19 Science Taskforce
Japan LIVE Dashboard	http://COVID-2019.live/en/ (accessed on 4 April 2023)	Yahoo Japan, Keio University and St Luke’s International University
US COVID Atlas	https://theuscovidatlas.org/ (accessed on 6 April 2023)	University of Chicago and partners.
COnVIDa	https://convida.inf.um.es/(no longer live) (accessed on 4 April 2023)	University of Murcia
Track COVID-19 in the U.S.	https://www.nytimes.com/interactive/2023/us/covid-cases.html (accessed on 4 April 2023)	The New York Times
COVID-19 Coronavirus Pandemic	https://www.worldometers.info/coronavirus/ (accessed on 4 April 2023)	Worldometer
Coronavirus Pandemic (COVID-19)	https://ourworldindata.org/coronavirus (accessed on 4 April 2023)	Our World in Data
Global COVID-19 Tracker	https://www.kff.org/coronavirus-covid-19/issue-brief/global-covid-19-tracker/ (accessed on 4 April 2023)	Kaiser Family Foundation
Coronavirus (COVID-19) in the UK	https://coronavirus.data.gov.uk/details/interactive-map/cases (accessed on 4 April 2023)	UK Government
COVID map: Coronavirus cases, deaths, vaccinations by country	https://www.bbc.co.uk/news/world-51235105 (accessed on 4 April 2023)	British Broadcasting Corporation
Web scraping tools **		
COVID-scraper		George Mason University and partners.
COVID-19 European Regional Tracker	https://asjadnaqvi.github.io/COVID19-European-Regional-Tracker/ (accessed on 25 March 2023)	European Union
CRISPER	https://crisper.net.au/ (accessed on 25 March 2023)	Australian National University and partners.
**Static data representations**		
COVID-19 in the UK	https://www.bbc.co.uk/news/uk-51768274 (accessed on 11 April 2023)	British Broadcasting Corporation
COVID hospitalisations: see the latest trend and current count	https://www.nbcnews.com/data-graphics/covid-hospitalizations-see-latest-trend-current-count-rcna61053 (accessed on 11 April 2023)	CNBC News

* Dashboards—online data platforms that have dynamic data display and have functions for data querying. ** These tools also have dashboards associated with them.

## Data Availability

Not applicable.
